# Sequence verification of synthetic DNA by assembly of sequencing reads

**DOI:** 10.1093/nar/gks908

**Published:** 2012-10-05

**Authors:** Mandy L. Wilson, Yizhi Cai, Regina Hanlon, Samantha Taylor, Bastien Chevreux, João C. Setubal, Brett M. Tyler, Jean Peccoud

**Affiliations:** ^1^Virginia Bioinformatics Institute, Virginia Tech, Washington Street MC 0477, Blacksburg, VA 24061, USA, ^2^DSM Nutritional Products Ltd., Department for Human Nutrition & Health, P.O. Box 2676, CH-4002 Basel, Switzerland, ^3^Center for Genome Research and Biocomputing, 3021 Agriculture and Life Sciences Building, Oregon State University, Corvallis, OR 97331-7303 and ^4^ICTAS Center for Systems Biology of Engineered Tissues, MC 0193 Virginia Tech, Blacksburg, VA 24061, USA

## Abstract

Gene synthesis attempts to assemble user-defined DNA sequences with base-level precision. Verifying the sequences of construction intermediates and the final product of a gene synthesis project is a critical part of the workflow, yet one that has received the least attention. Sequence validation is equally important for other kinds of curated clone collections. Ensuring that the physical sequence of a clone matches its published sequence is a common quality control step performed at least once over the course of a research project. GenoREAD is a web-based application that breaks the sequence verification process into two steps: the assembly of sequencing reads and the alignment of the resulting contig with a reference sequence. GenoREAD can determine if a clone matches its reference sequence. Its sophisticated reporting features help identify and troubleshoot problems that arise during the sequence verification process. GenoREAD has been experimentally validated on thousands of gene-sized constructs from an ORFeome project, and on longer sequences including whole plasmids and synthetic chromosomes. Comparing GenoREAD results with those from manual analysis of the sequencing data demonstrates that GenoREAD tends to be conservative in its diagnostic. GenoREAD is available at www.genoread.org.

## INTRODUCTION

Gene synthesis (1,2) is the process of manufacturing user-defined DNA sequences with base-level precision. The limitations of the chemistries used at different steps of the process require scientists to verify the physical sequence of the clones they produce at the different stages of the assembly process. The rapid development and commercial success of new high-throughput sequencing technologies calls for a careful analysis of the technology best suited to meet the sequence verification needs of gene synthesis operators. Difference of throughput, price structure and access to sequencing resources should be considered in relation to the gene synthesis facility throughput, nature of the sequences it produces and other technical and economic constraints. Since the verification of thousands of 1-kb building blocks is very different from the verification of a small number of 100-kb synthetic fragments, different sequencing technologies are used at different stages of synthetic genomics projects (3). In this fast-evolving landscape of sequencing technologies, Sanger sequencing still remains the most commonly used technology for sequence verification (4,5). While more expensive per base than newer sequencing technologies, Sanger is less expensive per run, making it more relevant to the job of clone-verification than it might be for a traditional genome-sized sequence verification project. Sanger remains the most cost-effective sequencing technology for most gene synthesis projects focused on assembling sequences that do not exceed a few kilobases in length.

The need to verify the sequence of clones and plasmids is not limited to gene synthesis; it also applies to any plasmid containing inserts with known sequences, such as clones from ORFeome collections, irrespective of the way the plasmid was assembled. It is common practice in molecular biology to verify the sequence of a plasmid prior to publication or submission to a community resource like Addgene (6), the Registry of Standard Biological Parts (7) or the DNASU repository (8). The value of integrating sequencing data in database applications to manage large collections of biological parts is now well recognized. Since efforts to verify the collection of clones distributed to the iGEM students demonstrated the need for systematic quality control of submissions to the Registry of Standard Biological Parts (7), users of this community resource have been encouraged to upload sequencing trace files. Each read is aligned with the part’s reference sequence, and the verification status of the clone is clearly displayed for each physical distribution in the Registry. Addgene provides a similar functionality to its users by sharing results of its own internal quality controls beside the sequence provided by the depositing scientist; however, the website does not provide users with tools to easily compare the two sequences. Sequence verification is therefore a critical part of the workflow of many projects in the life sciences, yet it is probably the one that has received the least attention. In GenoREAD, this process is composed of two successive steps. The physical sequence of a clone is first determined by analysing a number of sequencing traces. In a second step, the clone’s physical sequence is compared with the clone’s expected sequence, also called the *reference sequence*. Commercially available bioinformatics packages (VectorNTI, CLC Bio Workbench, Lasergene, and others) include algorithms necessary to perform this analysis. They can analyse trace files produced by sequencing instruments and align the output of the sequencing analysis with the reference sequence. They are often capable of batch processing large numbers of files in a single operation. However, none of these packages has a sequence verification feature. Even though sequence verification is a common problem, no commercial package provides a turnkey solution to this problem. The situation is similar with open-source packages like EMBOSS (9) or UGENE (10).

The lack of a sequence verification pipeline is more than just a convenience issue. Sequencing data are often manually compared to the plasmid reference sequence; this approach can be very time-consuming and prone to human error due to operator fatigue and a lack of rigorous analysis processes. Furthermore, the outcome of the sequence verification process is dependent on the algorithms selected at each step of the process and the parameters used when calling these algorithms. There is a real possibility for mistakes to be made during the sequence verification process when performing a manual analysis. These mistakes can lead to accepting a clone with undetected mutations, or they can lead to the rejection of perfectly acceptable clones that produced less than optimal sequencing data.

Since the purpose of the sequence verification step is to rule out discrepancies between a clone’s physical sequence and its expected sequence, it is critical to ensure that this step does not introduce new errors itself. This can be achieved by developing automated and validated sequence verification pipelines that can quickly and predictably analyse large collections of sequencing data with minimal user input. The Joint BioEnergy Institute Inventory of Composable Elements (JBEI-ICE) is an open-source software platform for managing collections of biological parts (11); it includes a feature called SequenceChecker that visually aligns sequencing data with the plasmid’s reference sequence with the goal of detecting discrepancies. SequenceChecker does not resolve conflicting reads nor does it determine the sequence verification status of the clone.

CloneQC is a web-based application (12) developed to automate the sequence verification of the large number of clones generated by the Synthetic Yeast 2.0 project (13,14). CloneQC allows users to upload two archives containing the trace files and the reference sequences. The sequencing reads are automatically matched with the corresponding reference sequence using BLAST (15). The forward and reverse reads are then more precisely aligned with the reference sequence using ClustalW (16). CloneQC then takes into consideration the alignment results along with the quality of the read to assign one of several quality statuses to the clone (Pass, Fail, Check, Fixable). CloneQC was the first tool to propose a rigorous algorithm to the verification of clones generated in the context of a large scale DNA synthesis operation. Its major limitation is that it cannot handle the verification of clones longer than the span of two Sanger sequencing reads, or about 2000 bp.

In this article, we describe GenoREAD, a new sequence verification application that breaks down the analysis process into two distinct steps: the assembly of the sequencing reads into a contig, and the alignment of the contig with the reference sequence. This approach allows GenoREAD to verify the sequence of short and long genetic constructs. The application workflow has been used on thousands of gene-sized constructs, as well as longer sequences, such as the complete sequences of plasmids and a 96-kb synthetic chromosome. GenoREAD provides sophisticated reporting capabilities that can help users uncover various sequencing verification problems. GenoREAD reports have been validated by comparing them to the results of a manual sequence verification process relying on desktop applications. This pipeline has been made available to the scientific community at www.genoread.org with the hope that it may facilitate the systematic verification of synthetic genetic constructs produced by gene synthesis and other cloning techniques.

## MATERIALS AND METHODS

### DNA sequencing

Glycerol stocks of bacterial clones from the Öomycete Effectorome Project (OEP) were sequenced by Beckman Genomics using the Single Pass Sequencing service. The OEP aims to clone nearly 1300 genes encoding RXLR effectors from Öomycete plant pathogens (17), using PCR (Tyler, B. unpublished data). Plasmids were purified by Solid Phase Reversible Immobilization (SPRI). DNA was sequenced by Sanger sequencing using BigDye Terminator v3.1. Post-reaction dye terminators were removed using Agencourt CleanSEQ. Sequence delineation was performed on an ABI PRISM 3730*x.* Sequencing traces were downloaded from the Beckman LIMS system as ab1 files.

### Bioinformatics workflow

**Figure 1.**
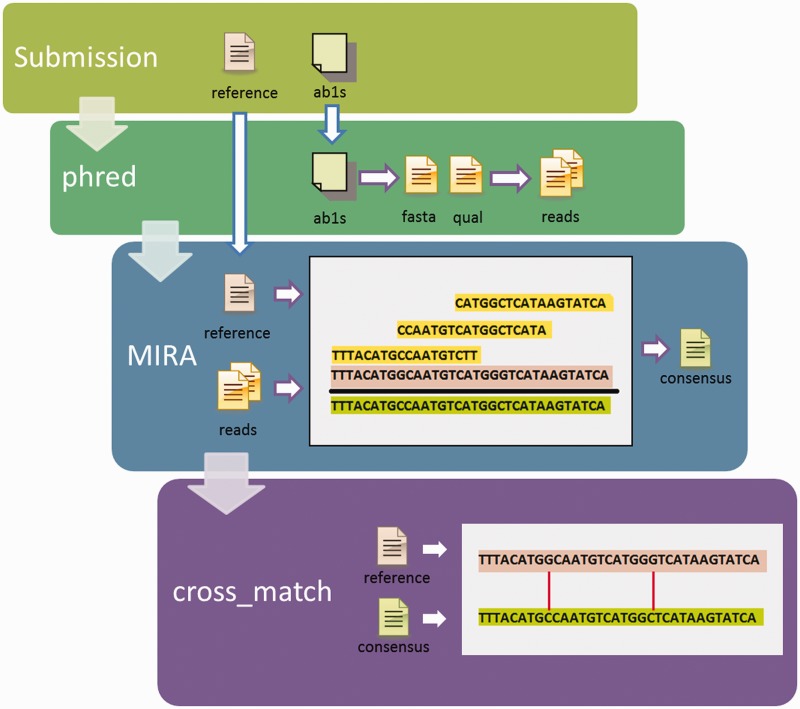
Data analysis workflow. GenoREAD iterates through a four-step process to verify trace files against a reference sequence. (i) Submission: the user submits their sequencing trace files and their reference sequence through the user interface. (ii) Phred converts the trace files into reads (sequences from the trace files in fasta format, and the quality scores in a separate.qual file). (iii) MIRA assembles the reads into a consensus sequence using the reference sequence as a backbone. (iv) Cross_match takes the consensus sequence from MIRA and aligns it against the reference sequence, delineating the differences between the two. Finally, the application presents the report to the user.

The GenoREAD sequence verification workflow is illustrated in Figure 1. It proceeds through three successive steps: base calling, assembly and alignment.

The base calling step is performed by Phred. Phred converts the sequencing trace files into a single read file in fasta format (18); it also creates a quality score file that assigns a score to each base pair (bp) in the read file based on the sequencing results. A quality score is an estimation of the probability of a base call error at a given position. Higher quality scores indicate more confidence in the assessment of the base called. When the chromatogram is ambiguous and the base called uncertain, then the quality scores generally fall below 20 (19).

The assembly step is performed by MIRA 3.4 (20)*,* an open-source assembler available from www.sourceforge.net/projects/mira-assembler/. MIRA uses the reference sequence to assemble the reads produced by Phred. MIRA produces a report that identifies inconsistencies between the reads, or between the reads and the reference sequence; MIRA also returns a *consensus* sequence, also called a contig. GenoREAD uses MIRA’s Sanger Assembly algorithm. It also takes advantage of the *mapping* assembly option, so the reference sequence is used as a backbone that guides the assembly process. Assembly by reference leads to longer contigs than *de novo* assembly. GenoREAD also relies on the MIRA *straindata* option; by assigning a separate strain name to the reads from the one used by the reference sequence, it is possible to identify a contig, which MIRA calls a *consensus* sequence, based solely on the reads without undue influence by the reference sequence. Without this option, the consensus sequence would try to fill in ‘gaps’ (uncovered sections of the reference sequence) with the backbone sequence, which could imply that the assembly was more successful than it really was. For clarity, the reference sequence is assigned a default quality score of 0 so the differences can be highlighted between the reads and the reference sequence in the assembly report. If there is a discrepancy between the reads, MIRA will usually rule in favor of the base with the highest quality score; however, if the quality scores are too close or equal, MIRA will return a non-DNA character (specifically, an IUPAC base) in place of a DNA base. This facilitates the identification of unresolvable discrepancies. Finally, GenoREAD uses MIRA’s default clipping behavior that trims the low-quality ends of the reads based on a minimum average quality score of 20 over a window of 30 consecutive base pairs.

The consensus sequence produced by MIRA is then aligned with the reference sequence using Cross_match, a sequence alignment utility distributed with the Phrap assembler (www.phrap.org). Alignment results provide the basis for the final Summary Report.

### User interface and software integration

GenoREAD is a web-based application currently hosted on a Dell PowerEdge R710 with 48 GB of RAM, 2 Xeon X5670@2.67 GHz (2x6 cores) processors and 4 2TB SAS Drives in a RAID 6 configuration. This server is hosted and managed by the Virginia Bioinformatics Institute Core Computational Facility.

The user interface is written in PHP 5. Software integration scripts are written in Perl. Graphics are produced using the GD Graphics Library.

A sequence verification process can take as little as a few seconds or it can take hours, depending on the number and complexity of the clones analysed. Upon completion of the process, results may be consulted using a web browser or downloaded as a zip file for future reference. Data analysis results are kept on the server for 24 h before they are purged from the system.

## RESULTS

### User interaction

The user interface gives users the option to verify either a single clone or a project consisting of multiple clones.

To verify a single clone, the user must provide the clone name, the reference sequence as a FASTA file and a zip file containing the trace files (ab1s) from sequencing the clone. Supplementary Data include a sample clone Dataset (Sample_Clone_Data.zip).

**Figure 2.**
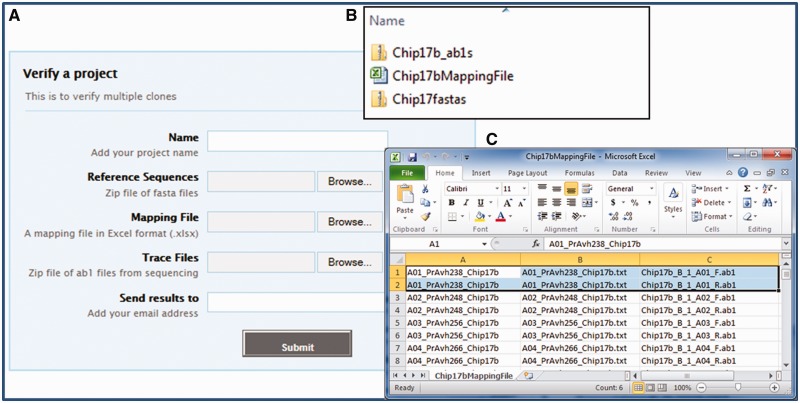
Submission of a verification project. (**A**) To submit a project (set of clones) for verification, the user needs to prepare three files in advance (**B**). The reference sequences must be contained in individual files that are compressed (zipped) together for review; a mapping file (in Excel 2010 (.xlsx) format) to indicate which reference and trace files should be used for each clone; and trace files compressed into a single zip file. The format for the mapping file (**C**) has the clone name in column A, the reference file name in column B and the trace file name in column C; there is no column header line. When there is more than one trace file for a given clone, every trace file gets its own row with the same clone and reference file name for all trace files for that clone (see rows 1 and 2 in the spreadsheet above).

In a high-throughput environment, clones are often sequenced in batches of 96 or 384. To verify the sequences of multiple clones grouped in a project corresponding to one or more plates, users must provide a project name (i.e. Chip20 Clones), a zip file containing all of the reference sequences in FASTA format, a mapping file in Excel 2010 (.xlsx) format and a zip file containing all the trace files for the project. The submission of a sequencing project is detailed in Figure 2. The Supplementary Data include a sample project dataset (Sample_Project_Data.zip).

Since the analysis of large projects can take minutes to hours, it is not always practical to wait for the results after submitting an analysis job. As a result, both the clone submission and project submission screens allow users to provide an email address which is used to send them a link to the sequence verification report. This email can also be used as a reference for retrieving the analysis report at a later time. In addition to viewing the analysis results on the GenoREAD website, users have the ability to download the components of the report (the input files as well as the output of the GenoREAD report) in zipped format.

### Sequence verification reports

The sequence verification of a clone generates several reports accessible from a single web page. The Summary Report summarizes the sequence verification results. A visual representation of the alignment of the contig and reference sequence is helpful for understanding alignment problems. The Assembly Report shows the assembly of the reads to the reference sequence. Discrepancies are highlighted in different colors depending on the type of discrepancy and whether it gets propagated to the contig or not; a key at the top of the report indicates the meaning of the various colors.

The web page also includes links to the reference sequence, the reads and the quality scores generated by Phred, graphs visualizing the quality of the trace files, the contig generated by MIRA and its quality scores and a graph visualizing the quality of the contig.

The essential analysis results are captured in the Summary Report, so most clones do not require the examination of the other reports. The Summary Report starts by reporting the clone name and the clone status. The clone status can be Pass, Fail or Review (see Table 1 for more information about the definition of these three statuses). Next, the Summary Report reports the length of the reference sequence and whether or not a contig was found. When the assembly was successful, the report includes the length of the contig and the average quality score of the contig, along with the positions in the contig where the quality score fell below 20. The Summary Report also reports the results of the alignment between the contig and the reference sequence, including a list of discrepancies in the alignment, their location and the quality score of that discrepancy in the contig.

**Table 1.** Criteria for determining the value of the status

If …	… the status will be:
The reference sequence is at least as long as the contig sequence AND There are no discrepancies in the alignment AND The alignment length is equal to the length of the reference sequence AND The average quality score is >20 AND There is only one alignment	Pass

MIRA found no contigs when assembling the reads against the reference sequence backbone	Fail

If the results fail to meet the requirements for the above two conditions	Review

The Supplementary Data include an archive called Clone_Data_Reports.zip that includes representative Datasets used to illustrate the different types of outcomes of the sequence verification workflow. The archive also includes a selection of relevant reports.

### Pass and Fail statuses

The two simplest outcomes are clones with a Pass or Fail status.

PsAvh463 is a clone from the OEP with a Pass status. The Summary Report prominently displays the Pass status of this clone. Generally, users would not need to look at any other report to have confidence that the physical sequence of this clone matches the reference sequence. A more detailed examination of the different reports shows that the forward and reverse traces resulted in two reads spanning the entire reference sequence with high-quality scores. There were no discrepancies, either between the reads, or between the reads and the reference sequence. As a result, MIRA was able to produce a high-quality contig that perfectly covered the entire reference sequence.

If read files cannot be assembled against the reference sequence, then the clone status is reported as Fail. OEP clone PrAvh248 illustrates this situation. In cases like this, there can be no alignment because there is no contig. In addition, the verification report will only include a brief Summary Report, the reference sequence, the sequences of the reads, the quality scores of the reads and the graphs of the quality scores per base pair for the reads. The graphs included in the report can help the user understand the reason for the failure to assemble the reads. One possibility is that the quality scores of the reads are consistently low (i.e. lower than an average score of 20 across a window length of 30 for the entire read). In this case, MIRA will drop the read entirely. If all of the reads are similarly poor, then this might suggest a sequencing failure, not a failure of the clone. If, on the other hand, the graphs show that the reads were of fairly high quality, then that would suggest the clone is not what it is supposed to be, either because of contamination or mislabeling problems.

Clones that cannot be classified as either a Pass or a Fail are reported as requiring Review to analyse the sequencing results. The following sections illustrate some of the problems that can lead to this situation.

### A clone with sequence discrepancies

**Figure 3.**
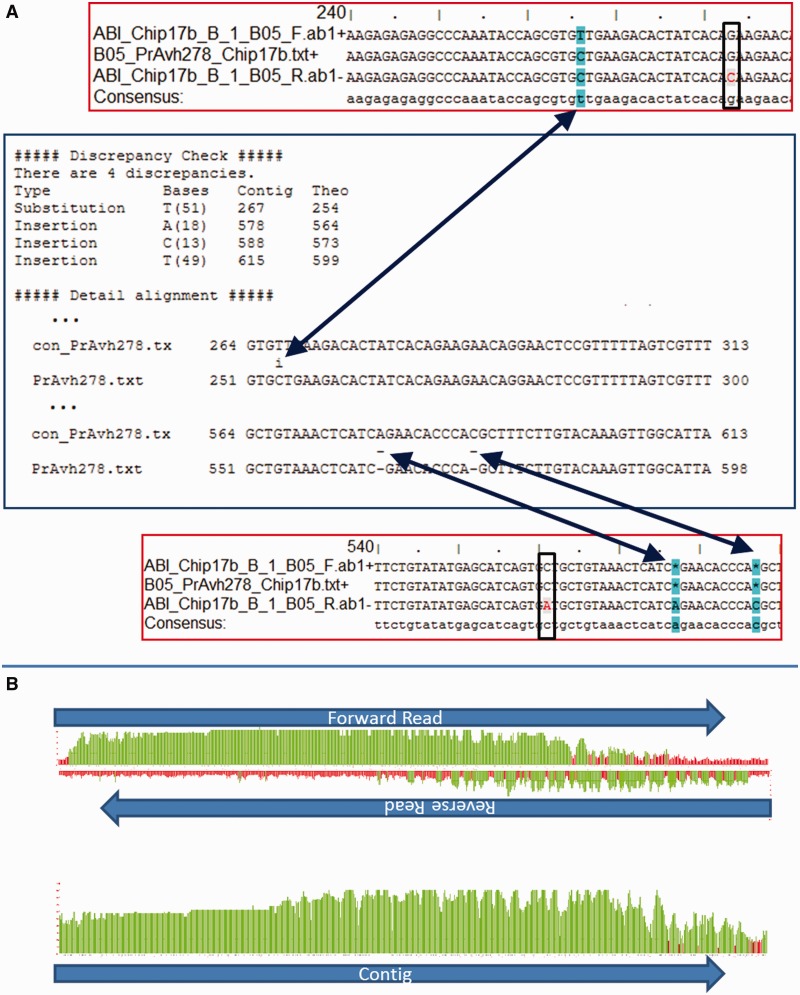
Sequence verification report. (**A**) This composite figure shows the relationships between the MIRA assembly report framed in red and the Cross_match alignment reports framed in blue for the PrAvh278 gene. The MIRA report includes four lines: a forward read, the reference sequence, the reverse read and the consensus sequence, or *contig*. The column in teal shows that at position 266, the forward read did not agree with the reference sequence, whereas the reverse read was consistent with the reference sequence. However, because the quality of the reverse read was lower than then quality of the forward read, MIRA called a T instead of the C of the reference sequence. At positions 283 and 562, the two reads were also inconsistent with each other, but, in these cases, the forward read was consistent with the reference sequence. Not surprisingly, Cross_match detects a substitution at position 266, but not at positions 282 and 561. Similarly, the report shows the presence of two deletions at positions 577 and 586. (**B**) Plotting the quality scores of the two reads clearly shows that the reverse read was not as good as the forward read. Yet, information from both reads was used by the assembler MIRA, which resulted in a fairly good quality for the contig except in the 5′ region of the gene where some of the bases have a quality score below 20.

A status of ‘Review (Discrepancies found)’ indicates the presence of one or more discrepancies between the contig and the reference sequence. PrAvh278—downloadable as a zip file from the Supplementary Data—illustrates how GenoREAD identifies discrepancies as shown in Figure 3.

The Discrepancy Check section of the Summary Report for PrAvh278 indicates that there are four discrepancies in this clone. The discrepancies also include the quality score of the contig at this location. For instance, the 254C>T substitution and the 599_600insT insertion have high-quality scores of 51 and 49, respectively, indicating a high level of confidence in the assembled sequence. The two other insertions, 564_565insA and 573_574insC, have lower quality scores of 18 and 13, respectively. These lower quality scores are an indication that the low quality of the reads at these locations made it difficult to resolve the sequence of the contig.

Even though the identification of discrepancies is performed by using Cross_match to compare the reference sequence and the contig, examining the assembly report produced by MIRA is useful to critically review the outcome of the assembly step. The four discrepancies identified by Cross_match are also visible on the assembly report, where they appear in teal color. It is interesting to observe that in all four cases, the two reads ABI_B05_F.ab1 and ABI_B05_R.ab1 gave inconsistent results. MIRA chose one read over the other based on their respective quality scores at these locations. It is also worth noting that the assembly report includes a number of places where the two reads did not agree, but MIRA called the base consistent with the reference sequence. The read that was ignored is highlighted by a red letter on a light gray background. Examination of the graphs of the read quality scores indicates that the reverse read quality is substandard, with a lot of regions reflecting quality scores below 20. Examining the quality scores of the resulting contig pinpoints areas where its quality drops below the 20 threshold. In these cases, it is important to keep in mind that there is a high probability for the contig to be inaccurate because of the poor quality of the individual reads. If a discrepancy is detected in a low-quality region, there is a chance that the clone does not have this discrepancy. Similarly, if the reference sequence and contig coincide in a low-quality region, it is impossible to rule out the presence of undetected discrepancies. These ambiguities could be resolved by adding another high-quality read and reanalysing the clone.

The Supplementary Data file named Sample_Project_Data.zip includes several clones, such as PrAvh266, where the two reads give consistent results with one another but differ from the reference sequence. Two good quality reads are generally sufficient to detect the presence of a discrepancy without uncertainty.

The Supplementary Data provide more information about the algorithm used by MIRA to resolve discrepancies between reads and assign quality scores to the contig (Supplementary Methods).

### Alignment problems

**Figure 4.**
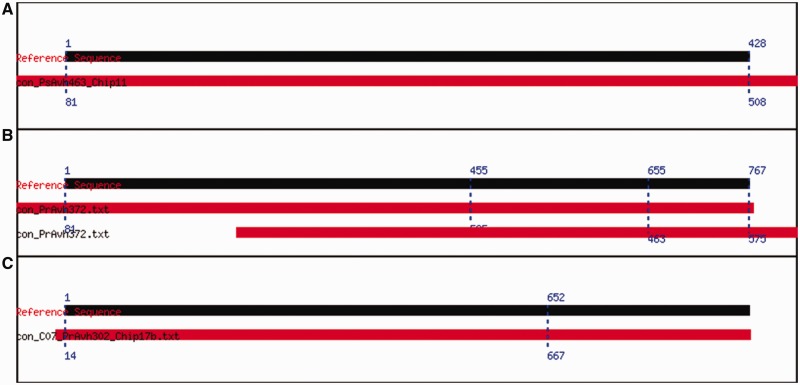
Different types of alignments. The alignment graphics try to capture how much of the reference sequence the contig covers and where they overlap. (**A**) Alignment of PsAvh463, a clone that matches its reference sequence. The black bar represents the reference sequence, the red one represents the contig and the dotted blue lines designate the positions of the boundaries of the alignment. (**B**) PrAvh372 illustrates a case where there are multiple alignments; neither of the alignments is complete. (**C**) PrAvh302 illustrates an incomplete alignment that spans the first 652 bases of the reference sequence.

In order to identify potential assembly problems, Cross_match aligns the contig assembled by MIRA with the reference sequence to identify discrepancies between the two. Ideally, this alignment would span the entire length of the reference sequence, but, under certain circumstances, Cross_match returns multiple alignments or a partial alignment, which is indicative of underlying problems. These alignment issues are described in more detail below, and compared in Figure 4.

*Multiple alignments* occur when Cross_match finds more than one possible alignment between the consensus sequence and the reference sequence. A multiple alignment does not always indicate a problem. Multiple alignments often occur, for example, if the contig contains a repeat. As long as one of the alignments runs the span of the reference sequence, there is probably no reason to reject the clone. Multiple alignments can also indicate a gap between the reads, or an especially ‘noisy’ section in the middle. For that reason, multiple alignments often walk hand-in-hand with mutations.

OEP clone PrAvh372 illustrates well the benefit of aligning the contig to the reference sequence. The Assembly Report shows a high concentration of discrepancies in the last 100 bases of the contig even though the quality of the reads and the contig are high. Cross_match uncovered the existence of two alignments, each a partial alignment at the ends of the reference sequence. Upon careful examination of the reference sequence, it appeared that it had an inexact repeat at the end which matched the second part of the contig exactly, while the contig did not include a repeat of any kind.

Incomplete alignments occur when only part of the reference sequence is covered by the contig. This typically happens with long sequences because reads can usually cover only between 800 and 1000 bp with adequately high-quality scores. A partial alignment can also indicate a major discrepancy between the reference sequence and the contig. This could happen, for instance, in situations where one fragment did not get inserted in a three-way ligation. Finally, partial alignments occur when one or more of the reads are excluded from the assembly because of poor quality scores. To determine if a read was dropped, the assembly report can be reviewed. The analysis of OEP clone PrAvh302, described in the Supplementary Data, is an example of a partial alignment resulting from the poor quality of one of the reads.

### Larger sequences

For the sake of simplicity, the previous examples focused on the verification of individual genes. One of the main benefits of using an assembler instead of simply aligning reads with the reference sequence is the possibility of verifying the sequences of larger constructs. We have used GenoREAD to verify the complete sequence of various plasmids.

**Figure 5.**
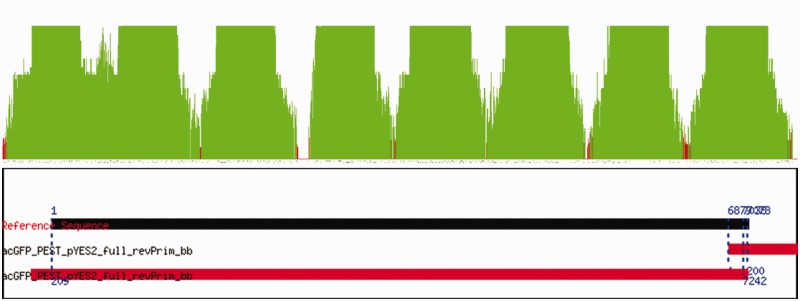
Complete sequence verification of a plasmid. (**A**) This 7 kb plasmid was sequenced using 16 sequencing primers. The quality score of the resulting contig highlights coverage issues. Regions with quality scores >20, illustrated in green, are separated by regions of quality scores <20, shown in red. There is even a region with a complete lack of coverage. (**B**) When sequencing circular DNA molecules, it is typical to observe two or three alignments with one of the alignments covering the entire reference sequence. The information in the graphics is complemented by the detailed alignment results included in the Summary Report.

The Supplementary Data include a set of sequencing data collected to verify the sequence of 7 yeast plasmids about 7 kb in size (Yeast_Plasmids_Data.zip). For each plasmid, we collected 16 trace files expected to cover the entire plasmid. Figure 5 illustrates how the quality scores of the contig can be used to quickly identify gaps in the coverage of the sequencing data that may result from sequencing primers which are sparsely distributed on the reference sequence. In these situations, it is often necessary to add more sequencing reads that use different primers specially designed to fill the gaps. Alternatively, the low quality of a read can create gaps that could be filled by collecting a better read from the same primer.

The circular nature of plasmids leads to alignment issues that call for careful manual review. It is common to observe two or three alignments, a long one and one or two short ones. As long as the long alignment covers the entire reference sequence, the plasmid can be accepted.

We also ran a test against a synthetic version of the right arm of yeast chromosome IX [8] to see how the application scaled up. The reference sequence was about 96 000 bp long, and had 929 trace files collected in 2008 and 2009. GenoREAD took only 3 min to produce the final report. The same dataset was also analysed with CLC Bio Workbench which produced three contigs using the default parameters of the assembly by reference algorithm. Even though it is unlikely that a construct of this size would still be sequenced using Sanger chemistry, this test case demonstrates that the application can gracefully handle complex sequencing datasets.

## DISCUSSION

Sequence-verification data are often analysed manually. In order to assess GenoREAD performance, Table 2 provides the results of a comparison of GenoREAD Status with the result of a manual analysis of the same sequencing data using a commercial desktop application (4Peaks from Mekentosj B.V.). It includes five sequencing plates representative of a larger set of more than 5900 sequencing reactions collected over a 12-month period from the OEP.

**Table 2.** Comparison of GenoREAD assessments to a Human (manual) review of sequencing results using 4Peaks

	Status	Manual	GenoREAD	Comments
Chip8	Pass	69	66	
	Review	10	13	
	Fail	17	17	

Chip9	Pass	52	47	
	Review	17	22	
	Fail	27	27	

Chip11	Pass	65	65	The technician interpreted one borderline case as a Fail, but GenoREAD marked it as a Review because MIRA found a very small contig.
	Review	15	16	
	Fail	16	15	

Chip12b	Pass	42	39	
	Review	39	42	
	Fail	15	15	

Effector Plate 5	Pass	68	67	The sequences on the Effector Plates had been confirmed once already, so only the forward reads were ordered for these plates.
	Review	23	24	
	Fail	5	5	

Overall, GenoREAD’s results are a little more conservative than the technician’s results. It should also be noted that the technician marked some of the results as successful after investigating the impact of discrepancies, whereas GenoREAD automatically classifies an alignment with discrepancies as requiring review.

Overall, GenoREAD results are slightly more conservative than the human review of sequencing data. This is appropriate, because borderline cases ought to be examined by a scientist who can use background information about the project goal to make educated decisions regarding the status of specific clones. For the most part, GenoREAD results and the human analysis were consistent with one another. In the cases where there was disagreement, GenoREAD never diagnosed a Pass where it should have called for a Review or Fail status.

There are a few limitations that should be considered when submitting a sequence to GenoREAD. GenoREAD does not handle circular constructs such as plasmids as thoroughly as might be desired. Alignment of circular sequences is notoriously difficult (21). In order to overcome this limitation, the design of the sequencing primer and the origin of the reference sequence should be consistent with one another to limit the extension of reads beyond the extremities of the reference sequence. In other words, the plasmid should be sequenced as if it was a linear DNA fragment. As a web-based application, there are also limitations on how much data can be submitted at once to the application. There is an upload limit of 100 Mbyte on the webserver, so if the combination of reference sequence(s) and compressed trace files exceeds this value, the verification will fail. Finally, data will only be available on the server for 24 h before it is removed to make room for other runs.

One cannot understate the importance of the contig quality score to the GenoREAD analysis. The Average Quality Score displayed in the Summary Report gives a global indication of the quality of the sequence verification. The list of positions with quality scores below 20 highlights problem areas that have not been verified with a high level of confidence. The more trace files that agree with each other, the higher the quality scores will tend to be. For example, if there is complete agreement between a single read file and the reference sequence, then the Status may still register as a Pass, but the average quality score may be around 60. With two trace files in complete agreement, the quality score would be higher, perhaps in the 70s or 80s, and with three trace files, the average quality score could be even higher as explained in the Supplementary Data. The assembler makes it possible to achieve a good quality score for contigs derived from multiple reads of marginal quality.

It would be desirable for the Status to take into consideration the quality of individual bases in the assembled sequence, but this is not yet the case for several reasons. The quality scores of the assembled sequence do not translate into a mathematical expression of the estimated error rate at each position. An extensive combination of experimental and computational efforts would be necessary to determine the probability of sequencing error with the contig quality score. Early on, the International Human Genome Consortium defined a quality objective for finished projects; sequences with <1 error in 10 000 bases with no gaps were deemed compliant with what became known as the Bermuda standard (22). Since then, the gold standard of genome assembly has been raised to <1 error in 100 000 bases (23,24).

In this context, it is surprising that the much simpler problem of sequence verification does not have any agreed upon quality standards (25). The specification of a quality standard for synthetic DNA sequences would greatly simplify the reporting of essential information for synthetic DNA sequences (26).

Furthermore, recent efforts to characterize the reliability of sequence-based assays in clinical microbiology uncovered a high probability of sequencing errors (27). The application of genomics tools to sequence verification is likely to help reduce the occurrence of sequencing verification errors. This is all the more necessary now that pioneering projects in synthetic genomics have stressed the need and difficulty of sequence verification at multiple steps in the assembly of synthetic genomes (3,13).

By relying on an established assembler like MIRA that can process data produced by more recent sequencing technologies, it will be possible to extend GenoREAD so that it can perform genome verification using massively parallel sequencing strategies. This capability could be used by synthetic genomics projects to verify the sequence of large assembly intermediates or the sequence of the completed genome (3,13). Sequence verification of genomes could also be useful outside of synthetic biology. Provided that the data analysis pipeline can be streamlined as for Sanger sequencing, one can envision that sequence verification of yeast strains and mutants may soon be a cost-effective alternative to PCR-based approaches (28). Recently, several authors described approaches using massively parallel sequencing technologies to identify and retrieve error-free oligonucleotides prior to their assembly (29,30). Looking forward, it is possible that the clear distinction between assembly and verification in DNA fabrication workflows will vanish.

## SUPPLEMENTARY DATA

Supplementary Data are available at NAR Online: Supplementary Methods and Supplementary Datasets.

## FUNDING

National Science Foundation (NSF) [EF-0850100, DBI-1241328 to J.P.]; Agriculture and Food Research Initiative of the National Institute of Food and Agriculture of the USDA [2010-65110-20764 to B.M.T.]. Funding for open access charge: NSF Award [DBI-1241328]; USDA [2010-65110-20764].

*Conflict of interest statement*. None declared.

## Supplementary Material

Supplementary Data
