# Supplementary material for: Sequence verification of synthetic DNA by assembly of sequencing reads
Source: Nucleic Acids Res. 2012 Oct 5;41(1):e25. doi: 10.1093/nar/gks908 (PMC3592409; doi:10.1093/nar/gks908)
Supplement: Supplementary Data [file supp_41_1_e25__index.html]

Sequence verification of synthetic DNA by assembly of sequencing reads — Supplementary Data 

# Sequence verification of synthetic DNA by assembly of sequencing reads

## Supplementary Data

files

**Files in this Data Supplement:**

- Supplementary Data - pdf file
- Supplementary Data - zip file
- Supplementary Data - zip file
- Supplementary Data - zip file
- Supplementary Data - zip file
