# Supplementary material for: Sequence verification of synthetic DNA by assembly of sequencing reads
Source: Nucleic Acids Res. 2012 Oct 5;41(1):e25. doi: 10.1093/nar/gks908 (PMC3592409; doi:10.1093/nar/gks908)
Supplement: Supplementary Data [file supp_gks908_nar-01874-met-h-2012-File011.pdf]

## MIRA's Algorithm for Defining the Consensus Sequence during a Mapping Assembly

During a mapping assembly, MIRA tries to assemble a series of reads to a target sequence, also called the *reference* sequence. This process is trivial when the reads are in perfect agreement with each other and the reference sequence, but often there are variations. This document explains how MIRA uses (and assigns) quality scores to resolve to a single consensus sequence.

Every alignment **L** of reads can be seen as succession of columns **C(i)**. Every column contains – for each base in this column – information about the read direction, base and quality. The description of the consensus algorithm in this text will use the following – slightly constructed – example:

**C= { +A40; +C20; +C20; +C20; +G20; -G20; *+T30*}**

which describes a column with 7 bases: 1 "A" in a read being in forward direction with a phred quality of 40; 3 "C" in forward reads, all with a phred quality 20; and 2 "G"; both with a phred quality of 20 but one being in a forward read and one in a reverse complement read; and finally a base T in a forward read with a quality of 30.

In addition to the criteria above, MIRA can be told that certain reads belong to different strains (or organisms or cell lines etc.). This information is considered both during the assembly process and when creating a consensus of an alignment; MIRA can either create the consensus only for a given strain or for all strains. For example, assume the user gave MIRA the information that the reference sequence (which is also represented as a read in the assembly) has the strain name "R" and all the other reads belong to strain "X"; the column from the example above can be rewritten like this:

**C= { +A40(x); +C20(x); +C20(x); +C20(x); +G20(x); -G20(x); *+T30*(r)}**.

The reference shows a "T" while the 6 mapped reads show either A, C, or G.

When creating the consensus, MIRA goes through the following process for each column position of an assembly:

1. Collect all reads belonging to the strain (or all strains) of interest. Following the example above, if MIRA is asked to create a consensus for strain "X", the column will be purged of reads not belonging to the searched strain, so

   **C= { +A40(x); +C20(x); +C20(x); +C20(x); +G20(x); -G20(x); *+T30*(r)}**

   becomes

   **C= { +A40(x); +C20(x); +C20(x); +C20(x); +G20(x); -G20(x)}**

   as the base T belonged to a read from the "R" strain and not to the "X" strain. For the following steps in this example, the strain information is not of interest anymore and left out from the column representation:

**C= { +A40; +C20; +C20; +C20; +G20; -G20}**

2. In a process called "Down weighting bases", MIRA adjusts the weights of the phred quality score based on the location of the column in the assembly. First, MIRA considers the column's position with respect to the vector sequence; for each read of a column, MIRA checks to see if the base is within 10 base pairs of the end of the read identified as the sequencing vector sequence. If so, the Phred quality score for this base is set to either two times the distance of the position to the end of read, or to the originally assigned quality score, whichever is lower. This effectively implements a quality ramp-up, ramp-down function towards clipping points of vector sequences. The reason behind this preprocessing of the reads (vector or adaptor removal), is that sometimes clipping is done too conservatively and leaves a couple of vector / adaptor bases in a read. Down weighting bases there implicitly gives a higher weight for bases in other reads which are not at the end of a read, and helps lowers the impact of wrong clipping.
3. Next, MIRA does down-weighting of bases near the ends of reads. If the base is not within ten base pairs of a sequencing vector sequence, check whether it was near the end of the read itself (distance shared with contig endmark_read_exclusion_area (-CO:emea) parameter); if so, set the Phred quality score for this base to either two times the distance of the position to the end of the read or to the originally assigned quality score, whichever is lower. The reason MIRA performs this assessment is because base calling and quality scoring programs like phred tend to make the most errors towards the start and the end of a read.
4. Now MIRA repartitions the values into base subsets, making a subset for A, one for C, one for G, one for T, and one for gaps ("*"). The subsets for the above example would be:

   **C= { A{+40}; C{+20; +20; +20}; G{+20; -20}; T{}; *{}}**

   Note that as the groups for "T" and "*" are empty, they are dropped altogether.
5. MIRA repartition then repartitions each base subset into base-directed subsets; in other words, split the subsets into forward reads and reverse reads. Continuing the example above:

   **C= { A{+{40}; -{}}; C{+{20; 20; 20}; -{}}; G{+{20}; -{20}}}**

   Note that the groups A- and C- being empty, they are dropped:

   **C= { A{+{40}}; C{+{20; 20; 20}}; G{+{20}; -{20}}}**

6. For each directed subset, calculate a directed group quality by taking the highest quality and add to that 1/10th of the second highest quality. For the example above:

   **A+{40}:** Since there is only one quality, the directed group quality is therefore the quality of the base: Q(a+)=40

**C+{20; 20; 20}:** In this case, there are three qualities. The highest quality is 20, and the second highest is 20, so the directed group quality is therefore Q(c+)= 20 + 20/10 = 22

**G+{20}:** There is only one quality, the directed group quality is therefore the quality of the base: Q(g+)=20

**G-{20}:** There is only one quality, so the directed group quality is therefore the quality of the base: Q(g-)=20

MIRA has used this computation scheme since the earliest 0.x versions (1998 onward) due to the observation that while the bases of different reads are independent measurements, some sequencing technologies have known problems with certain sequences and often produce the same erroneous base calls in different reads. Therefore, the error probabilities of bases in reads pointing into the same direction cannot be seen as independent, and cannot be multiplied, or added in the logarithmic quality space represented by phred values. However, seeing a base called more than once should give a little bit more confidence in the base than seeing it only once, hence the 1/10 factor in quality space.

7. Add together the two directed qualities to form a base group quality. In the example above:

   **Q(a)= Q(a+) + Q(a-) = 40 + 0 = 40**
   **Q(c)= Q(c+) + Q(c-) = 22 + 0 = 22**
   **Q(g)= Q(g+) + Q(g-) = 20 + 20 = 40**
   **Q(t) = 0**
   **Q(*)=0**

   In contrast to reads pointing in the same direction, base qualities from reads pointing in the reverse complement direction can be seen as mostly independent, as error patterns are unlikely to affect both directions at once. Therefore, error probabilities can be multiplied, or the base qualities added.

8. Having done this, MIRA checks to see whether there is only one group with quality. If so, e.g. only Q(a)=30, things are easy: that's the base "A". However, if there is a conflict like above where more than one base has a quality prediction (Q(a)>0 && Q(c)>0 && Q(g)>0), the system needs to decide whether to take only one group or take two (or more) groups (making it a IUPAC base, unless one of the groups is a gap). In a case like this, MIRA looks again at how the group qualities were built: how many reads compose that group, whether forward and reverse directions are present. The rules are technology dependent; since GenoREAD is using Sanger, there would ideally be group quality for both directions, at least three reads for that base, and a minimum quality value of 35. If this is not the case, the minimum target quality value is decreased in decrements of 5 until one or several groups are found. If only one group is found things are again easy: that's the base. In the example above:

9. **Q(a) = 40** is good from the quality perspective, but has only the + direction covered
   **Q(c) = 22** is below 35

**Q(g) = 40** is good from the quality perspective and has both directions covered. This is better than Q(a) having the same quality, but only one direction.
The consensus is therefore “G” with a quality value of 40. No ties, no IUPAC base.

If two or more groups are found to tie, then the consensus sequence is assigned the IUPAC base of the found groups (ie, A or G = R). If one of the groups is a gap, then IUPAC could not represent that and it becomes a numerical shoot-out between the number of reads with gaps and the number of reads in groups without gaps, winner takes all.

As example for a tie: if the column had shown

**C= { +A35; -A10; +C20; +C20; +C20; +G20; -G20}**

this would have resulted in Q(a)= 35 + 10/10= 36; Q(c) = 22 and Q(g) = 40, making the called base a tie between A (quality 36 and in both directions) and G (quality 40 and in both directions): a “R” with a quality of (Q(a)+Q(g))/2 = (36+40) / 2 = 38

The consensus algorithm described above does have a couple of weaknesses, namely:

- Gaps are weakened by the fact that they cannot be represented in IUPAC and thus may have less impact than they should.
- The starting quality of 35 in step 8 is used as a cut off instead as a sliding window start. This leads to the slightly unnerving possibility that ties between groups having qualities of 40 and 36 (quality distance 4, see example above) are represented as IUPAC bases, while ties with, e.g. 37 and 33 (also quality distance 4) let the group with the quality of 37 win completely and show no IUPAC but a base. This is a known weakness.

Important note: the algorithm described here applies just to the determination of a consensus with one sequencing technology (Sanger). Determination of the consensus sequence with several technologies in hybrid de-novo or mapping assemblies is similar, but the determination is done for each technology separately and with an additional decision layer for resolving ties between technologies.
