# Supplementary material for: Sequence verification of synthetic DNA by assembly of sequencing reads
Source: Nucleic Acids Res. 2012 Oct 5;41(1):e25. doi: 10.1093/nar/gks908 (PMC3592409; doi:10.1093/nar/gks908)
Supplement: Supplementary Data [file supp_gks908_nar-01874-met-h-2012-File010.zip › Clone_Data_Reports/Clone_Data_Reports/C_Discrepancies_PrAvh278_Data_Report/Reports/PrAvh278_assembly_report.htm]

Project PrAvh278\_2012-07-23\_112555\_assemble 


# Tag legend

|  |  |
| --- | --- |
| = FCDS; | Feature CDS (coding sequence) |
| = FtRN; | tRNA |
| = FrRN; | rRNA |
| = Fm-R; | misc. RNA |
| = MISM; | Mismatch (discrepancy) between reads and consensus |
| = SRMx; | Strong Repeat Marker Base set by MIRA |
| = WRMx; | Weak Repeat Marker Base set by MIRA |
| = SROx; | SNP inteR Organism (Read/Consensus) set by MIRA |
| = SAOx; | SNP intrA Organism (Read/Consensus) set by MIRA |
| = SIOx; | SNP Inter- and intra-Organism (Read/Consensus) set by MIRA |
| = MCVc; | Missing CoVerage in Consensus (set by MIRA) |
| = POLY; | Poly-A signal |
| = EDxD; | Delete operation set by EdIt |
| = EDxI; | Insert operation set by EdIt |
| = EDxC; | Change operation set by EdIt |
| = IUPAC; | IUPAC base (shows only in HTML output) |


# PrAvh278.txt\_bb

## Statistics

To be reworked!

## Sequence:

|  |  |
| --- | --- |
| 0 | |    .    |    .    |    .    |    .    |    .    |    . |
| ABI\_B05\_F.ab1+ | TTATAATGCCAACTTTGTACAAAAAAGCAGGCTCCCAGGAGGCCACCATGGCCCTGAGCA |
| PrAvh278.txt+ | TTTGTACAAAAAAGCAGGCTCCCAGGAGGCCACCATGGCCCTGAGCA |
| Consensus: | ttataatgccaactttgtacaaaaaagcaggctcccaggaggccaccatggccctgagca |

|  |  |
| --- | --- |
| 60 | |    .    |    .    |    .    |    .    |    .    |    . |
| ABI\_B05\_F.ab1+ | TGAAGAAAACCGATGACAACAGCCGTGCCCAGGTTCACTCCATTCGCCACGGTGACCACG |
| PrAvh278.txt+ | TGAAGAAAACCGATGACAACAGCCGTGCCCAGGTTCACTCCATTCGCCACGGTGACCACG |
| Consensus: | tgaagaaaaccgatgacaacagccgtgcccaggttcactccattcgccacggtgaccacg |

|  |  |
| --- | --- |
| 120 | |    .    |    .    |    .    |    .    |    .    |    . |
| ABI\_B05\_F.ab1+ | TAAGCCTCGGGCAGTCCGAGGAAAGAGCTCATCACGCGGAATTATCAACAATTCGCTTGC |
| PrAvh278.txt+ | TAAGCCTCGGGCAGTCCGAGGAAAGAGCTCATCACGCGGAATTATCAACAATTCGCTTGC |
| Consensus: | taagcctcgggcagtccgaggaaagagctcatcacgcggaattatcaacaattcgcttgc |

|  |  |
| --- | --- |
| 180 | |    .    |    .    |    .    |    .    |    .    |    . |
| ABI\_B05\_F.ab1+ | GGGATCTACAGAGCGTCGAGGCCACGAGTGCTGCGAAGCCCATGAACGACGTCCAAAGTG |
| PrAvh278.txt+ | GGGATCTACAGAGCGTCGAGGCCACGAGTGCTGCGAAGCCCATGAACGACGTCCAAAGTG |
| ABI\_B05\_R.ab1- | GGCCACGAGTGCTGCGAAGCCCATTAACAACGTCCAAAGCG |
| Consensus: | gggatctacagagcgtcgaggccacgagtgctgcgaagcccatgaacgacgtccaaagtg |

|  |  |
| --- | --- |
| 240 | |    .    |    .    |    .    |    .    |    .    |    . |
| ABI\_B05\_F.ab1+ | AAGAGAGAGGCCCAAATACCAGCGTGTTGAAGACACTATCACAGAAGAACAGGAACTCCG |
| PrAvh278.txt+ | AAGAGAGAGGCCCAAATACCAGCGTGCTGAAGACACTATCACAGAAGAACAGGAACTCCG |
| ABI\_B05\_R.ab1- | AAGAGAGAGGCCCAAATACCAGCGTGCTGAAGACACTATCACACAAGAACAGGAACTCCG |
| Consensus: | aagagagaggcccaaataccagcgtgttgaagacactatcacagaagaacaggaactccg |

|  |  |
| --- | --- |
| 300 | |    .    |    .    |    .    |    .    |    .    |    . |
| ABI\_B05\_F.ab1+ | TTTTTAGTCGTTTCAAAGGACTATTGGGAAAGAATCCAACCAAGCTCACCCAAAAGAATG |
| PrAvh278.txt+ | TTTTTAGTCGTTTCAAAGGACTATTGGGAAAGAATCCAACCAAGCTCACCCAAAAGAATG |
| ABI\_B05\_R.ab1- | TTTTTAGTCGTTTCACAGGACTATTGGGAAAGAATCCAACCAAGCTCACCCAAAAGAATG |
| Consensus: | tttttagtcgtttcaaaggactattgggaaagaatccaaccaagctcacccaaaagaatg |

|  |  |
| --- | --- |
| 360 | |    .    |    .    |    .    |    .    |    .    |    . |
| ABI\_B05\_F.ab1+ | TCGAGAACATCAAGGAGAACAGCAAACTCAAAGGGCTACTGAGCAAAAACCCGTCGACGC |
| PrAvh278.txt+ | TCGAGAACATCAAGGAGAACAGCAAACTCAAAGGGCTACTGAGCAAAAACCCGTCGACGC |
| ABI\_B05\_R.ab1- | TCGAGAACATCAAGGAGAACAGCAAACTCAAAGGGCTAGTGAGCAAAAACCCGTCGACGC |
| Consensus: | tcgagaacatcaaggagaacagcaaactcaaagggctactgagcaaaaacccgtcgacgc |

|  |  |
| --- | --- |
| 420 | |    .    |    .    |    .    |    .    |    .    |    . |
| ABI\_B05\_F.ab1+ | TCAGCAAAAAGAGTGCTGGCAGGCTTGGGGGGTATTTGGCCAAGAATGGCTTGACGCAGA |
| PrAvh278.txt+ | TCAGCAAAAAGAGTGCTGGCAGGCTTGGGGGGTATTTGGCCAAGAATGGCTTGACGCAGA |
| ABI\_B05\_R.ab1- | TCAGCACAAAGAGTGCTGGCAGGCTTGGGGGGTATTTGGCCAAGAATGGCTTGAAGCAGA |
| Consensus: | tcagcaaaaagagtgctggcaggcttggggggtatttggccaagaatggcttgacgcaga |

|  |  |
| --- | --- |
| 480 | |    .    |    .    |    .    |    .    |    .    |    . |
| ABI\_B05\_F.ab1+ | TCTATTCACGTGCTGTGATTGCACTGTTTGGAGCAGGTGCATTGGCAGCTTTGATCTATG |
| PrAvh278.txt+ | TCTATTCACGTGCTGTGATTGCACTGTTTGGAGCAGGTGCATTGGCAGCTTTGATCTATG |
| ABI\_B05\_R.ab1- | TCTATTCACGTGCTGTGATTGCACTGTTTGGAGCAGGTGCATTGGCAGCTTTGATGTATG |
| Consensus: | tctattcacgtgctgtgattgcactgtttggagcaggtgcattggcagctttgatctatg |

|  |  |
| --- | --- |
| 540 | |    .    |    .    |    .    |    .    |    .    |    . |
| ABI\_B05\_F.ab1+ | TTCTGTATATGAGCATCAGTGCTGCTGTAAACTCATC\*GAACACCCA\*GCTTTCTTGTAC |
| PrAvh278.txt+ | TTCTGTATATGAGCATCAGTGCTGCTGTAAACTCATC\*GAACACCCA\*GCTTTCTTGTAC |
| ABI\_B05\_R.ab1- | TTCTGTATATGAGCATCAGTGATGCTGTAAACTCATCAGAACACCCACGCTCTCTTGTAC |
| Consensus: | ttctgtatatgagcatcagtgctgctgtaaactcatcagaacacccacgctttcttgtac |

|  |  |
| --- | --- |
| 600 | |    .    |    .    |    .    |    .    |    .    |    . |
| ABI\_B05\_F.ab1+ | AAAGTTGGCATTATTAAGAAAGCATTTGCTTATCAATTTGGTGCAACGAACAGGTCACTA |
| PrAvh278.txt+ | AAAGTTGGCATTA\*TAAGAAAGCA |
| ABI\_B05\_R.ab1- | AAAGTAGGCATTA\*TAAGAAAGCA\*TTGCTTATCAATTTGTTGCAACGAACAGGTCACTA |
| Consensus: | aaagttggcatta\*taagaaagcatttgcttatcaatttgttgcaacgaacaggtcacta |

|  |  |
| --- | --- |
| 660 | |    .    |    .    |    .    |    .    |    .    |    . |
| ABI\_B05\_F.ab1+ | TCAGTCAAAAT |
| ABI\_B05\_R.ab1- | TCAGTCAAAAT |
| Consensus: | tcagtcaaaat |
