# Supplementary material for: Sequence verification of synthetic DNA by assembly of sequencing reads
Source: Nucleic Acids Res. 2012 Oct 5;41(1):e25. doi: 10.1093/nar/gks908 (PMC3592409; doi:10.1093/nar/gks908)
Supplement: Supplementary Data [file supp_gks908_nar-01874-met-h-2012-File010.zip › Clone_Data_Reports/Clone_Data_Reports/D_MultipleAlignments_PrAvh372_Data_Report/Reports/PrAvh372_assembly_report.htm]

Project PrAvh372\_2012-07-23\_115012\_assemble 


# Tag legend

|  |  |
| --- | --- |
| = FCDS; | Feature CDS (coding sequence) |
| = FtRN; | tRNA |
| = FrRN; | rRNA |
| = Fm-R; | misc. RNA |
| = MISM; | Mismatch (discrepancy) between reads and consensus |
| = SRMx; | Strong Repeat Marker Base set by MIRA |
| = WRMx; | Weak Repeat Marker Base set by MIRA |
| = SROx; | SNP inteR Organism (Read/Consensus) set by MIRA |
| = SAOx; | SNP intrA Organism (Read/Consensus) set by MIRA |
| = SIOx; | SNP Inter- and intra-Organism (Read/Consensus) set by MIRA |
| = MCVc; | Missing CoVerage in Consensus (set by MIRA) |
| = POLY; | Poly-A signal |
| = EDxD; | Delete operation set by EdIt |
| = EDxI; | Insert operation set by EdIt |
| = EDxC; | Change operation set by EdIt |
| = IUPAC; | IUPAC base (shows only in HTML output) |


# PrAvh372.txt\_bb

## Statistics

To be reworked!

## Sequence:

|  |  |
| --- | --- |
| 0 | |    .    |    .    |    .    |    .    |    .    |    . |
| ABI\_Chip17b\_B\_1\_H11\_R.ab1- | CAAATAATGATTTTATTTTGACTGATAGTGACCTGTTCGTTGCAACAAATTGATGAGCAA |
| ABI\_Chip17b\_B\_1\_H11\_F.ab1+ | ACAAATTGATGAGCAA |
| Consensus: | caaataatgattttattttgactgatagtgacctgttcgttgcaacaaattgatgagcaa |

|  |  |
| --- | --- |
| 60 | |    .    |    .    |    .    |    .    |    .    |    . |
| ABI\_Chip17b\_B\_1\_H11\_R.ab1- | TGCTTTTTTATAATGCCAACTTTGTACAAAAAAGCAGGCTCCCAGGAGGCCACCATGGCC |
| ABI\_Chip17b\_B\_1\_H11\_F.ab1+ | TGCTTTTTTATAATGCCAACTTTGTACAAAAAAGCAGGCTCCCAGGAGGCCACCATGGCC |
| PrAvh372.txt+ | TTTGTACAAAAAAGCAGGCTCCCAGGAGGCCACCATGGCC |
| Consensus: | tgcttttttataatgccaactttgtacaaaaaagcaggctcccaggaggccaccatggcc |

|  |  |
| --- | --- |
| 120 | |    .    |    .    |    .    |    .    |    .    |    . |
| ABI\_Chip17b\_B\_1\_H11\_R.ab1- | GCAGTGACGACCTCAAATGCAAGAAGCTCTGCTTTGATGAAAACGGCTCGTTCGACCACG |
| ABI\_Chip17b\_B\_1\_H11\_F.ab1+ | GCAGTGACGACCTCAAATGCAAGAAGCTCTGCTTTGATGAAAACGGCTCGTTCGACCACG |
| PrAvh372.txt+ | GCAGTGACGACCTCAAATGCAAGAAGCTCTGCTTTGATGAAAACGGCTCGTTCGACCACG |
| Consensus: | gcagtgacgacctcaaatgcaagaagctctgctttgatgaaaacggctcgttcgaccacg |

|  |  |
| --- | --- |
| 180 | |    .    |    .    |    .    |    .    |    .    |    . |
| ABI\_Chip17b\_B\_1\_H11\_R.ab1- | CTGGGTGGGAATGAAATACCCTACCAGCGCTTTCTGAGGACTCAACGCTCCGGCGATAAC |
| ABI\_Chip17b\_B\_1\_H11\_F.ab1+ | CTGGGTGGGAATGAAATACCCTACCAGCGCTTTCTGAGGACTCAACGCTCCGGCGATAAC |
| PrAvh372.txt+ | CTGGGTGGGAATGAAATACCCTACCAGCGCTTTCTGAGGACTCAACGCTCCGGCGATAAC |
| Consensus: | ctgggtgggaatgaaataccctaccagcgctttctgaggactcaacgctccggcgataac |

|  |  |
| --- | --- |
| 240 | |    .    |    .    |    .    |    .    |    .    |    . |
| ABI\_Chip17b\_B\_1\_H11\_R.ab1- | GATGGTGAAGAACGAGGGTTCGACGTCTCTAAGTTACAAACGTGGCTGCTGCCGAAGGGC |
| ABI\_Chip17b\_B\_1\_H11\_F.ab1+ | GATGGTGAAGAACGAGGGTTCGACGTCTCTAAGTTACAAACGTGGCTGCTGCCGAAGGGC |
| PrAvh372.txt+ | GATGGTGAAGAACGAGGGTTCGACGTCTCTAAGTTACAAACGTGGCTGCTGCCGAAGGGC |
| Consensus: | gatggtgaagaacgagggttcgacgtctctaagttacaaacgtggctgctgccgaagggc |

|  |  |
| --- | --- |
| 300 | |    .    |    .    |    .    |    .    |    .    |    . |
| ABI\_Chip17b\_B\_1\_H11\_R.ab1- | TTCCAACAAAAACTCGAGGGTTGGCTCCAAAAGGGAAAACCCGCGAAAGAAGTCTTTGAT |
| ABI\_Chip17b\_B\_1\_H11\_F.ab1+ | TTCCAACAAAAACTCGAGGGTTGGCTCCAAAAGGGAAAACCCGCGAAAGAAGTCTTTGAT |
| PrAvh372.txt+ | TTCCAACAAAAACTCGAGGGTTGGCTCCAAAAGGGAAAACCCGCGAAAGAAGTCTTTGAT |
| Consensus: | ttccaacaaaaactcgagggttggctccaaaagggaaaacccgcgaaagaagtctttgat |

|  |  |
| --- | --- |
| 360 | |    .    |    .    |    .    |    .    |    .    |    . |
| ABI\_Chip17b\_B\_1\_H11\_R.ab1- | GGATTACAACTCACCAAATTTGGGGATGAGCTACTTGTCAACCCCACGTTTATCGCTTGG |
| ABI\_Chip17b\_B\_1\_H11\_F.ab1+ | GGATTACAACTCACCAAATTTGGGGATGAGCTACTTGTCAACCCCACGTTTATCGCTTGG |
| PrAvh372.txt+ | GGATTACAACTCACCAAATTTGGGGATGAGCTACTTGTCAACCCCACGTTTATCGCTTGG |
| Consensus: | ggattacaactcaccaaatttggggatgagctacttgtcaaccccacgtttatcgcttgg |

|  |  |
| --- | --- |
| 420 | |    .    |    .    |    .    |    .    |    .    |    . |
| ABI\_Chip17b\_B\_1\_H11\_R.ab1- | GTCAAATATGTGGACGATTTGAGTGCAAAGTACCCCGGGAAGGCGGCGTCGACGATTCCA |
| ABI\_Chip17b\_B\_1\_H11\_F.ab1+ | GTCAAATATGTGGACGATTTGAGTGCAAAGTACCCCGGGAAGGCGGCGTCGACGATTCCA |
| PrAvh372.txt+ | GTCAAATATGTGGACGATTTGAGTGCAAAGTACCCCGGGAAGGCGGCGTCGACGATTCCA |
| Consensus: | gtcaaatatgtggacgatttgagtgcaaagtaccccgggaaggcggcgtcgacgattcca |

|  |  |
| --- | --- |
| 480 | |    .    |    .    |    .    |    .    |    .    |    . |
| ABI\_Chip17b\_B\_1\_H11\_R.ab1- | ACATTGGCCGCTGAGTATGGAGACGAAGCATTGTTCAAAATGCTGGAGGCAGCGAACCCA |
| ABI\_Chip17b\_B\_1\_H11\_F.ab1+ | ACATTGGCCGCTGAGTATGGAGACGAAGCATTGTTCAAAATGCTGGAGGCAGCGAACCCA |
| PrAvh372.txt+ | ACATTGGCCGCTGAGTATGGAGACGAAGCATTGTTCAAAATGCTGGAGGCAGCG\*ATGAA |
| Consensus: | acattggccgctgagtatggagacgaagcattgttcaaaatgctggaggcagcgaaccca |

|  |  |
| --- | --- |
| 540 | |    .    |    .    |    .    |    .    |    .    |    . |
| ABI\_Chip17b\_B\_1\_H11\_R.ab1- | GCTTTCTTGTA\*\*CAAAGT\*TGGC\*ATTATAAGAAAGCATTGCTTATCAATTTGTTGCAA |
| ABI\_Chip17b\_B\_1\_H11\_F.ab1+ | GCTTTCTTGTA\*\*CAAAGT\*TGGC\*ATTATAAGAAAGCATTGCTTATCAATTTGTTGCAA |
| PrAvh372.txt+ | GGTTCCAAGTACGCAGAGTCTGGCTACCA\*AATTACG\*A\*\*GCTGATCAAATTG\*AGCAT |
| Consensus: | gctttcttgta\*\*caaagt\*tggc\*attataagaaagcattgcttatcaatttgttgcaa |

|  |  |
| --- | --- |
| 600 | |    .    |    .    |    .    |    .    |    .    |    . |
| ABI\_Chip17b\_B\_1\_H11\_R.ab1- | CGAACAGGTCACTATCAGTCAAAATAA |
| ABI\_Chip17b\_B\_1\_H11\_F.ab1+ | CGAACAGGTCACTATCAGTCAAAAT\*AAAATCATTATTTG |
| PrAvh372.txt+ | TGGGCA\*GTCA\*T\*T\*GGAAAACCTCCGGGTGGCGTTTTGAAGATATTCGCTGCAGAAAA |
| Consensus: | cgaacaggtcactatcagtcaaaat\*aaaatcattatttgaagatattcgctgcagaaaa |

|  |  |
| --- | --- |
| 660 | |    .    |    .    |    .    |    .    |    .    |    . |
| PrAvh372.txt+ | GACAGCGAGTACTAAGTCAATCTTTGCAGACTGGGTCAAGTATGTCGACAATTTTAACGT |
| Consensus: | gacagcgagtactaagtcaatctttgcagactgggtcaagtatgtcgacaattttaacgt |

|  |  |
| --- | --- |
| 720 | |    .    |    .    |    .    |    .    |    .    |    . |
| PrAvh372.txt+ | AAAACACCCCGACAAGCAAATATCGCGGCGTCGACGATTCCAACATTGGCCGCTGAGTAT |
| Consensus: | aaaacaccccgacaagcaaatatcgcggcgtcgacgattccaacattggccgctgagtat |

|  |  |
| --- | --- |
| 780 | |    .    |    .    |    .    |    .    |    .    |    . |
| PrAvh372.txt+ | GGAGACGAAGCATTGTTCAAAATGCTGGAGGCAGCGAACCCAGCTTTCTTGTACAAAGTT |
| Consensus: | ggagacgaagcattgttcaaaatgctggaggcagcgaacccagctttcttgtacaaagtt |

|  |  |
| --- | --- |
| 840 | |    .    |    .    |    .    |    .    |    .    |    . |
| PrAvh372.txt+ | GGCATTATAAGAAAGCA |
| Consensus: | ggcattataagaaagca |
