# Supplementary material for: Sequence verification of synthetic DNA by assembly of sequencing reads
Source: Nucleic Acids Res. 2012 Oct 5;41(1):e25. doi: 10.1093/nar/gks908 (PMC3592409; doi:10.1093/nar/gks908)
Supplement: Supplementary Data [file supp_gks908_nar-01874-met-h-2012-File010.zip › Clone_Data_Reports/Clone_Data_Reports/E_PartialAlignment_PrAvh302_Data_Report/Reports/PrAvh302_assembly_report.htm]

Project PrAvh302\_2012-07-23\_115437\_assemble 


# Tag legend

|  |  |
| --- | --- |
| = FCDS; | Feature CDS (coding sequence) |
| = FtRN; | tRNA |
| = FrRN; | rRNA |
| = Fm-R; | misc. RNA |
| = MISM; | Mismatch (discrepancy) between reads and consensus |
| = SRMx; | Strong Repeat Marker Base set by MIRA |
| = WRMx; | Weak Repeat Marker Base set by MIRA |
| = SROx; | SNP inteR Organism (Read/Consensus) set by MIRA |
| = SAOx; | SNP intrA Organism (Read/Consensus) set by MIRA |
| = SIOx; | SNP Inter- and intra-Organism (Read/Consensus) set by MIRA |
| = MCVc; | Missing CoVerage in Consensus (set by MIRA) |
| = POLY; | Poly-A signal |
| = EDxD; | Delete operation set by EdIt |
| = EDxI; | Insert operation set by EdIt |
| = EDxC; | Change operation set by EdIt |
| = IUPAC; | IUPAC base (shows only in HTML output) |


# C07\_PrAvh302\_Chip17b.txt\_bb

## Statistics

To be reworked!

## Sequence:

|  |  |
| --- | --- |
| 0 | |    .    |    .    |    .    |    .    |    .    |    . |
| ABI\_C07\_F.ab1+ | TTATAATGCCAACTTTGTACAAAAAAGCAGGCTCCCAGGAGGCCACCATGGCCATGGATA |
| C07\_PrAvh302\_Chip17b.txt+ | TTTGTACAAAAAAGCAGGCTCCCAGGAGGCCACCATGGCCATGGATA |
| Consensus: | ttataatgccaactttgtacaaaaaagcaggctcccaggaggccaccatggccatggata |

|  |  |
| --- | --- |
| 60 | |    .    |    .    |    .    |    .    |    .    |    . |
| ABI\_C07\_F.ab1+ | AACGACCACTGGTGGTCAATGAATGGCCAGCCTCTGACAGTTCCCAATTCGCTCTGAACG |
| C07\_PrAvh302\_Chip17b.txt+ | AACGACCACTGGTGGTCAATGAATGGCCAGCCTCTGACAGTTCCCAATTCGCTCTGAACG |
| Consensus: | aacgaccactggtggtcaatgaatggccagcctctgacagttcccaattcgctctgaacg |

|  |  |
| --- | --- |
| 120 | |    .    |    .    |    .    |    .    |    .    |    . |
| ABI\_C07\_F.ab1+ | CCACGCATTGCTTGTGTTCCTGTATCCCATGGGAGTTTCCCAAGTTCTATTGGTCACCGT |
| C07\_PrAvh302\_Chip17b.txt+ | CCACGCATTGCTTGTG\*TCCTGTATCCCATGGGAG\*TTCCCAAGTTCTATTGGTCACCGT |
| Consensus: | ccacgcattgcttgtgttcctgtatcccatgggagtttcccaagttctattggtcaccgt |

|  |  |
| --- | --- |
| 180 | |    .    |    .    |    .    |    .    |    .    |    . |
| ABI\_C07\_F.ab1+ | CGTTGCCCTCTTCGCCTGTAGCGATTTTGCTGGAGTCGTAGAAGCCTACCCGATCAAGCG |
| C07\_PrAvh302\_Chip17b.txt+ | CGTTGCCCTCTTCGCCTGTAGCGATTTTGCTGGAGTCGTAGAAGCCTACCCGATCAAGCG |
| Consensus: | cgttgccctcttcgcctgtagcgattttgctggagtcgtagaagcctacccgatcaagcg |

|  |  |
| --- | --- |
| 240 | |    .    |    .    |    .    |    .    |    .    |    . |
| ABI\_C07\_F.ab1+ | CTCAGCGGTGACTTCGACTGACACTCTCACTCATGGCCACTCAATCGTCGCGGGAGGAAG |
| C07\_PrAvh302\_Chip17b.txt+ | CTCAGCGGTGACTTCGACTGACACTCTCACTCATGGCCACTCAATCGTCGCGGGAGGAAG |
| Consensus: | ctcagcggtgacttcgactgacactctcactcatggccactcaatcgtcgcgggaggaag |

|  |  |
| --- | --- |
| 300 | |    .    |    .    |    .    |    .    |    .    |    . |
| ABI\_C07\_F.ab1+ | ATCGCTGCGTGTGGAACAAACAACAAACGTCGAAGACGGCGACTCCGAGGAGAGGGTGAA |
| C07\_PrAvh302\_Chip17b.txt+ | ATCGCTGCGTGTGGAACAAACAACAAACGTCGAAGACGGCGACTCCGAGGAGAGGGTGAA |
| Consensus: | atcgctgcgtgtggaacaaacaacaaacgtcgaagacggcgactccgaggagagggtgaa |

|  |  |
| --- | --- |
| 360 | |    .    |    .    |    .    |    .    |    .    |    . |
| ABI\_C07\_F.ab1+ | TTTGTGGACAAAAATTTCCAAGCCTGTGAAACTCAAATTGTGGCGCTGGAGAGGCAAATC |
| C07\_PrAvh302\_Chip17b.txt+ | TTTGTGGACAAAAATTTCCAAGCCTGTGAAACTCAAATTGTGGCGCTGGAGAGGCAAATC |
| Consensus: | tttgtggacaaaaatttccaagcctgtgaaactcaaattgtggcgctggagaggcaaatc |

|  |  |
| --- | --- |
| 420 | |    .    |    .    |    .    |    .    |    .    |    . |
| ABI\_C07\_F.ab1+ | AGACGACTTTGTCAAAAGCGAGCTCGGAATGAAGGCCTTGTCAGGAGCAGCGCTTAAAGC |
| C07\_PrAvh302\_Chip17b.txt+ | AGACGACTTTGTCAAAAGCGAGCTCGGAATGAAGGCCTTGTCAGGAGCAGCGCTTAAAGC |
| Consensus: | agacgactttgtcaaaagcgagctcggaatgaaggccttgtcaggagcagcgcttaaagc |

|  |  |
| --- | --- |
| 480 | |    .    |    .    |    .    |    .    |    .    |    . |
| ABI\_C07\_F.ab1+ | GCATCCGAACTACCGTACCTACCTGAACTTCAAACTCGGCAAGTGGTACCGAAACGAGAA |
| C07\_PrAvh302\_Chip17b.txt+ | GCATCCGAACTACCGTACCTACCTGAACTTCAAACTCGGCAAGTGGTACCGAAACGAGAA |
| Consensus: | gcatccgaactaccgtacctacctgaacttcaaactcggcaagtggtaccgaaacgagaa |

|  |  |
| --- | --- |
| 540 | |    .    |    .    |    .    |    .    |    .    |    . |
| ABI\_C07\_F.ab1+ | GCTTACCACGCTTGGTGCCTGGGAGCGCTTGAAGCTGAGTGACATTCCTGTTGATAAGCT |
| C07\_PrAvh302\_Chip17b.txt+ | GCTTACCACGCTTGGTGCCTGGGAGCGCTTGAAGCTGAGTGACATTCCTGTTGATAAGCT |
| Consensus: | gcttaccacgcttggtgcctgggagcgcttgaagctgagtgacattcctgttgataagct |

|  |  |
| --- | --- |
| 600 | |    .    |    .    |    .    |    .    |    .    |    . |
| ABI\_C07\_F.ab1+ | TCGCAGTACCGACGCCTACATGACCTACGTCCGCTATGTAAATATCTTTGACGATAATGC |
| C07\_PrAvh302\_Chip17b.txt+ | TCGCAGTACCGACGCCTACATGACCTACGTCCGCTATGTAAATATCTTTGACGATAATGC |
| Consensus: | tcgcagtaccgacgcctacatgacctacgtccgctatgtaaatatctttgacgataatgc |

|  |  |
| --- | --- |
| 660 | |    .    |    .    |    .    |    .    |    .    |    . |
| ABI\_C07\_F.ab1+ | TATGCGA |
| C07\_PrAvh302\_Chip17b.txt+ | TATGCGAGCAGTGGAGGCCAAGCGCAAGACGCCCCCTCTAGTGAGTACTAATTTTGATAG |
| Consensus: | tatgcgagcagtggaggccaagcgcaagacgccccctctagtgagtactaattttgatag |

|  |  |
| --- | --- |
| 720 | |    .    |    .    |    .    |    .    |    .    |    . |
| C07\_PrAvh302\_Chip17b.txt+ | CGAGGCGTCCAAGCTGGAGATGTTCGAGAGAGCGTGGATTTGGGGAGTGACTGGAAGATC |
| Consensus: | cgaggcgtccaagctggagatgttcgagagagcgtggatttggggagtgactggaagatc |

|  |  |
| --- | --- |
| 780 | |    .    |    .    |    .    |    .    |    .    |    . |
| C07\_PrAvh302\_Chip17b.txt+ | AAACGATTACGTTGTAACGGCGCTGGGAATGAGGGGGGTATCAGGAGAGGTGCTGATAAA |
| Consensus: | aaacgattacgttgtaacggcgctgggaatgaggggggtatcaggagaggtgctgataaa |

|  |  |
| --- | --- |
| 840 | |    .    |    .    |    .    |    .    |    .    |    . |
| C07\_PrAvh302\_Chip17b.txt+ | TCACGCAAACTCCCGATACTACGTGAAATATGCGAGGACAAAATTGAATCTAATGGGAAC |
| Consensus: | tcacgcaaactcccgatactacgtgaaatatgcgaggacaaaattgaatctaatgggaac |

|  |  |
| --- | --- |
| 900 | |    .    |    .    |    .    |    .    |    .    |    . |
| C07\_PrAvh302\_Chip17b.txt+ | CCAGCTTTCTTGTACAAAGTTGGCATTATAAGAAAGCA |
| Consensus: | ccagctttcttgtacaaagttggcattataagaaagca |
