# Supplementary figures and images for: Sequence verification of synthetic DNA by assembly of sequencing reads
Source: Nucleic Acids Res. 2012 Oct 5;41(1):e25. doi: 10.1093/nar/gks908 (PMC3592409; doi:10.1093/nar/gks908)

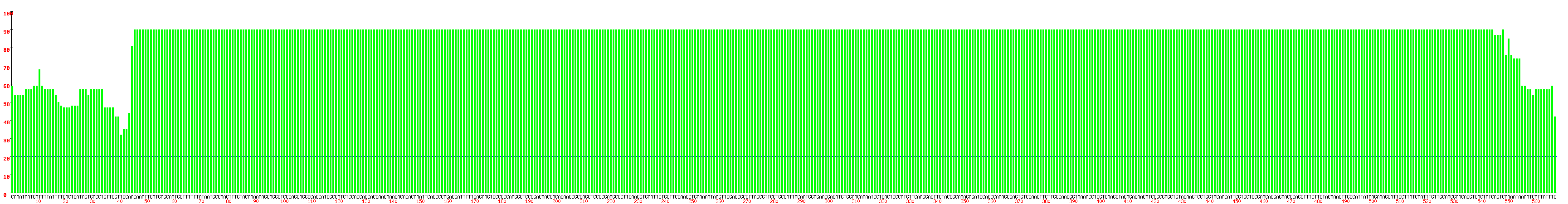

Supplement: Supplementary Data [file supp_gks908_nar-01874-met-h-2012-File010.zip › Clone_Data_Reports/Clone_Data_Reports/A_Pass_PsAvh463_Data_Report/Report/contigs_con_PsAvh463_Chip11.png]

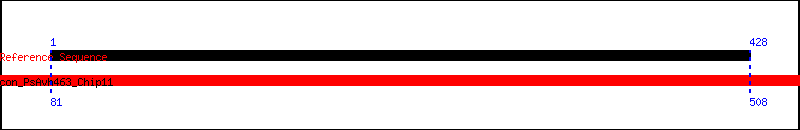

Supplement: Supplementary Data [file supp_gks908_nar-01874-met-h-2012-File010.zip › Clone_Data_Reports/Clone_Data_Reports/A_Pass_PsAvh463_Data_Report/Report/Pass_PsAvh463_alignment.png]

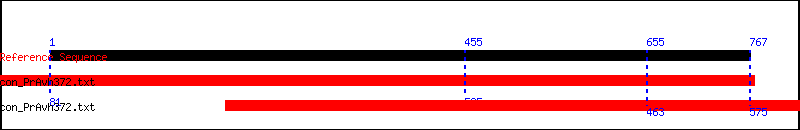

Supplement: Supplementary Data [file supp_gks908_nar-01874-met-h-2012-File010.zip › Clone_Data_Reports/Clone_Data_Reports/D_MultipleAlignments_PrAvh372_Data_Report/Reports/PrAvh372_alignment.png]

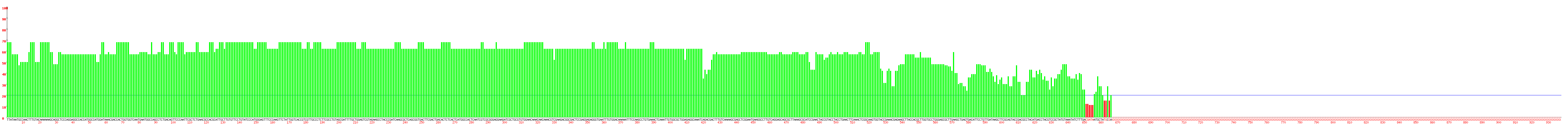

Supplement: Supplementary Data [file supp_gks908_nar-01874-met-h-2012-File010.zip › Clone_Data_Reports/Clone_Data_Reports/E_PartialAlignment_PrAvh302_Data_Report/Reports/contigs_PrAvh302.png]

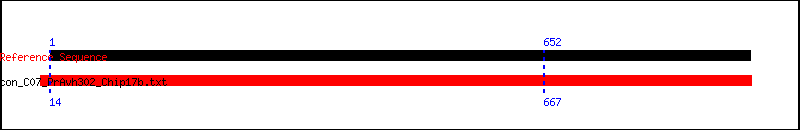

Supplement: Supplementary Data [file supp_gks908_nar-01874-met-h-2012-File010.zip › Clone_Data_Reports/Clone_Data_Reports/E_PartialAlignment_PrAvh302_Data_Report/Reports/PrAvh302_alignment.png]
